# Involvement of Kynurenine Metabolism in Bipolar Disorder: An Updated Review

**DOI:** 10.3389/fpsyt.2021.677039

**Published:** 2021-07-26

**Authors:** Peifen Zhang, Huimin Huang, Xingle Gao, Jiajun Jiang, Caixi Xi, Lingling Wu, Yaoyang Fu, Jianbo Lai, Shaohua Hu

**Affiliations:** ^1^Department of Psychiatry, The First Affiliated Hospital, Zhejiang University School of Medicine, Hangzhou, China; ^2^Wenzhou Medical University, Wenzhou, China; ^3^The Key Laboratory of Mental Disorder's Management in Zhejiang Province, Hangzhou, China; ^4^Brain Research Institute of Zhejiang University, Hangzhou, China

**Keywords:** bipolar disorder, kynurenine pathway, immune, pathogenesis, treatment

## Abstract

Bipolar disorder (BD) is a severe affective disorder, mainly characterized by alternative depressive and manic or hypomanic episodes, yet the pathogenesis of BD has not been fully elucidated. Recent researches have implicated the altered kynurenine (KYN) metabolism involved in the neurobiology of BD. Excessive activation of the immune system also occurs in patients with BD, which further accelerates the KYN pathway for tryptophan metabolism. Changes of the KYN metabolites have effects on neuronal receptors and are involved in neuroendocrine transmissions. Interactions between KYN metabolism and the immune system may contribute to the neuropathogenesis of BD. Various studies have shown that alterations of the KYN metabolites were associated with mood, psychotic symptoms, and cognitive functions in patients with BD. In this review, we briefly introduce the KYN pathway and describe the immune dysregulation in BD as well as their interactions. We then focus on the research advances on the KYN metabolism in BD, which hold promise for identifying novel treatment targets in patients stricken with this disorder.

## Introduction

Bipolar disorder (BD) is a chronic and recurrent mental disorder, characterized by alternating episodes of depression and mania or hypomania, along with significant cognitive impairments (1). It is estimated that ~1% population worldwide is affected and the lifetime prevalence ranges from 0.6 to 2.4% (2, 3). The onset age of BD is mainly during the adolescence (4). Most patients with BD firstly present with depressive episodes during the illness course and its differential diagnosis with major depressive disorder (MDD) remains clinically challenging. Due to the lack of typical markers, the confirmed diagnosis of BD is largely based on clinical experience and may be delayed for 5 or even 10 years (5, 6), thus contributing to the unfavorable prognosis and suicidal risks (7). However, the pathogenesis of BD has not been fully elucidated yet.

Previous studies showed that immune dysfunctions, such as elevated inflammatory and proinflammatory factors, were accompanied by the progression of BD or even occurred before the onset of illness (8, 9). As indicated by two landmark studies, administration of lipopolysaccharide (LPS) failed to elicit depressive-like behaviors when the stimulation of the kynurenine (KYN) pathway was blocked, despite that the levels of proinflammatory cytokines were elevated (10, 11). Immune activation could contribute to the metabolism of tryptophan (TRP) by shifting catabolic routes toward the KYN pathway (12). Proinflammatory responses and the mood status-related immune stimulation also trigger the KYN pathway in BD (13), predominantly via activating the key enzymes, such as indoleamine 2,3-dioxygenase (IDO) (14). Subsequently, various metabolites act on downstream receptors, including the N-methyl-D aspartate (NMDA) receptor and non-competitively α7 nicotinic acetylcholine receptor (α7nAChR) in the brain, thus modulating neuroendocrine transmission and brain functions (15). Therefore, the KYN pathway may be a bridge that mediates immune and neuroendocrine dysregulations in the pathogenesis of BD.

The purpose of this review is to outline the KYN pathway, the immune dysfunction, and their interactions in BD, which may emerge as novel treatment targets for this intractable disease. In this review, we firstly introduce the KYN pathway in regard to its metabolism, relationship with immune dysfunction and involvement in the pathogenesis of BD. We also summarize the current findings of the alterations of the KYN pathway metabolites and their correlations with the clinical symptoms of BD as well as the potential targets aiming at the KYN pathway. Finally, we discuss the current dilemma, and potential measures to tackle with this disease.

## The Kyn Pathway Metabolism

**Figure 1:**
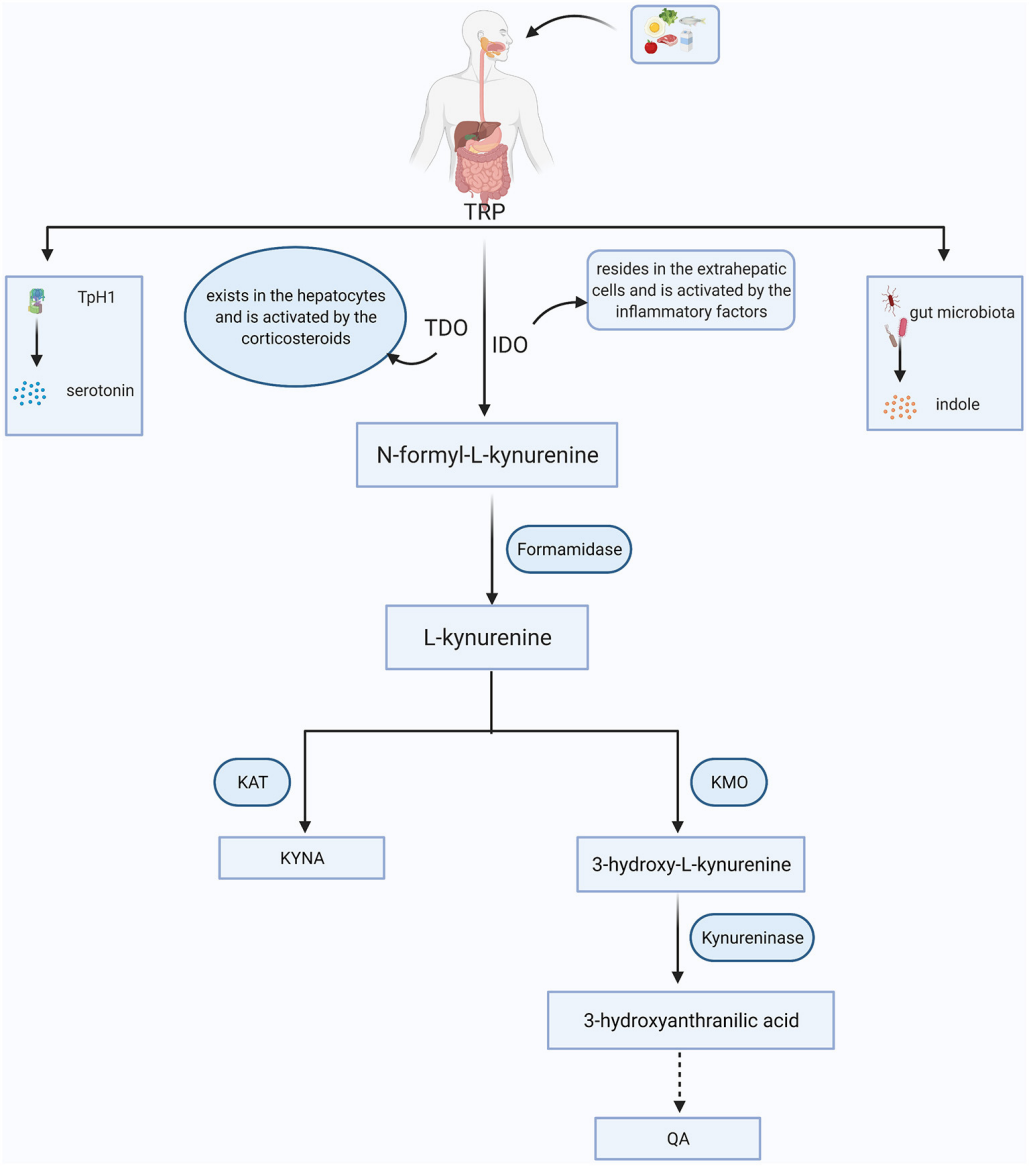
The simplified KYN metabolism pathways in general. Tryptophan (TRP) is obtained from food. Besides being converted into serotonin through tryptophan hydroxylase 1 (TpH1) and indoles via gut microorganism, more than 90% of TRP is broken down to the N-formyl-L-kynurenine with the aid of tryptophan 2,3-dioxygenase (TDO) or indoleamine 2,3-dioxygenase (IDO). N-formyl-L-kynurenine is further degraded to kynurenine (KYN) via formamidase and the KYN can be converted into kynurenic acid (KYNA) and quinolinic acid (QA) mainly depending on the kynurenine aminotransferase (KAT) and kynurenine 3-monooxygenase (KMO), respectively.

As an essential amino acid in the human body, TRP is exclusively obtained by dietary intake. In addition to serotonin and indoles, ~95% of TRP is massively diverted into the primary intermediate metabolite- N-formyl-L-kynurenine, which can be further catabolized into KYN via two key enzymes, tryptophan 2,3-dioxygenase (TDO) and IDO (12). TDO exists in the intra-hepatic tissues (16), while IDO mainly resides in the extrahepatic tissues, especially in the brain and immune cells, such as astrocytes, microglia, macrophages and monocytes (17). Once KYN is produced, it is mainly converted into quinolinic acid (QA) and kynurenic acid (KYNA) along with two distinct metabolic branches. KYN can be decomposed into 3-hydroxykynurenine (3-HK) by kynurenine 3-monooxygenase (KMO). 3-HK is the precursor of 3-hydroxyanthranilic acid (3-HAA), which is further metabolized into an agonist of the NMDA receptor, QA. Along with another branch, KYN is metabolized into KYNA via kynurenine aminotransferase (KAT) enzyme, and KYNA is regarded as an antagonist of the NMDA receptor with neuroprotective effects (18). In physiological states, these two branches coordinate with each other and maintain normal brain functions (Figure 1).

**Figure 2:**
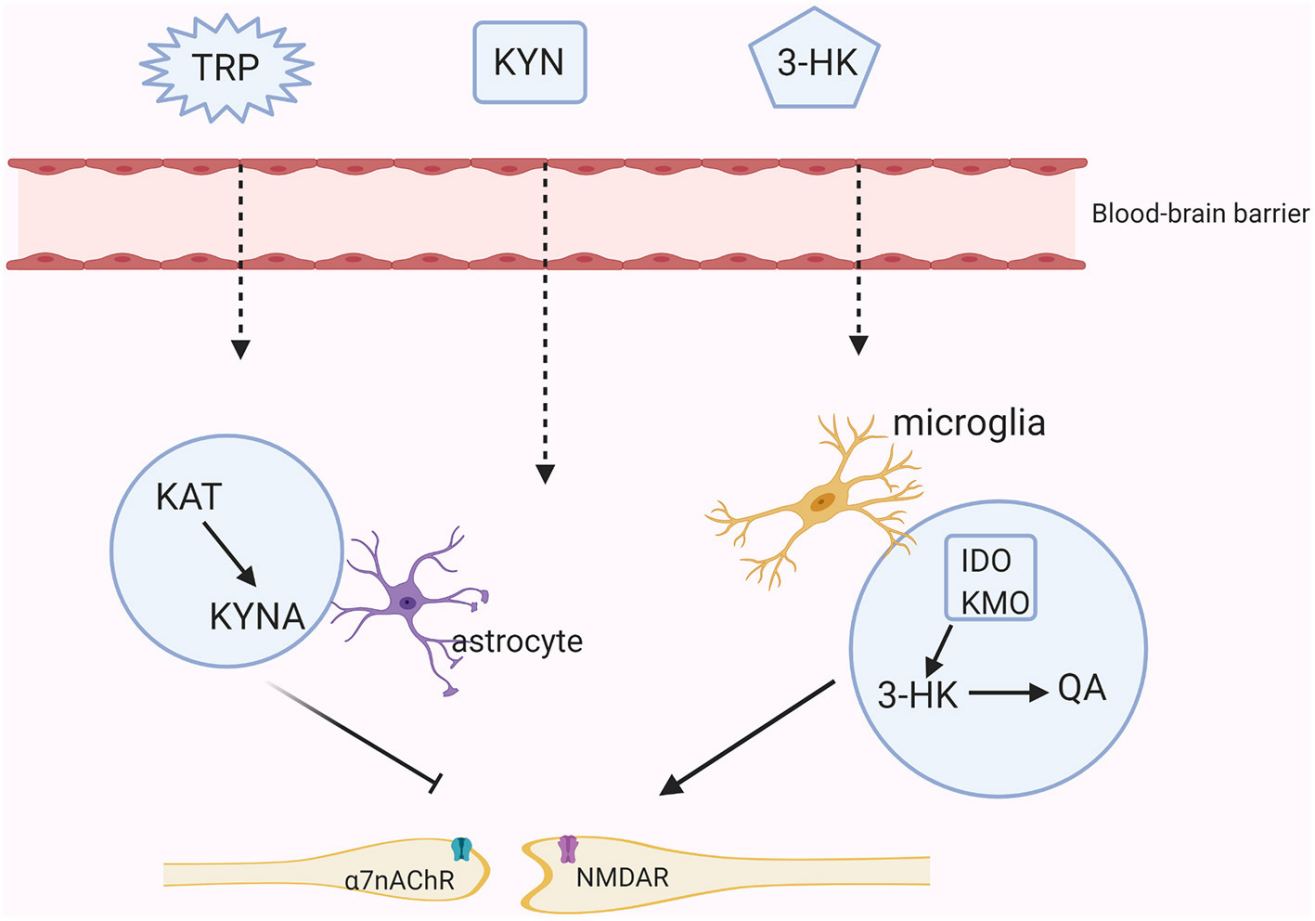
The KYN pathway in the brain. Tryptophan (TRP), kynurenine (KYN), and 3-hydroxykynurenine (3-HK) all can cross the brain-blood barrier (BBB). In astrocytes, KYN is mainly metabolized into the neuroprotective metabolite- kynurenic acid (KYNA) through the kynurenine aminotransferase (KAT). KYNA inhibits the activities of the N-methyl-D aspartate (NMDA) receptor and the non-competitively α7-nicotinic receptor (α7nAChR). While in microglia, KYN is further metabolized into an agonist of the NMDA receptor-quinolinic acid (QA) through indoleamine the 2,3-dioxygenase (IDO) and kynurenine monooxygenase (KMO).

Although KYNA and QA poorly cross the blood-brain barrier (BBB) under physiological processes, they also exist in the central nervous system (CNS) because nearly 60% of KYN and TRP can go through the BBB with the assistance of amino acid transporters (19). Moreover, these two main branches of the KYN metabolism are segregated in different cerebral glial cells. Specifically, QA is produced in microglia whereas KYNA is synthesized in astrocytes (20). The production of 3-HK generates free radicals and thus can induce oxidative stress. Overproduction of QA activates the NMDA receptor, causing synaptic dysregulation, oxidative stress and even neuronal apoptosis (21). KYNA acts on the NMDA receptor and the α7nAChR to regulate glutamate, synaptic plasticity and cognitive functions [(22); Figure 2]. Meanwhile, the normal functions of the KYN metabolites depend on the neuronal signals although existing evidence has shown that the KYN metabolism in the cerebra mainly occurs in the glial cells rather than in the neurons (23).

## The Immune-Kyn-Neuroendocrine Network in BD

### Immune Dysregulation in BD

Accumulating evidence has demonstrated that the immune system is dysregulated in BD. For example, the risk of developing BD is increased in patients experiencing autoimmune diseases (24). A high level of interleukin-6 (IL-6) was predominantly observed in mania, tumor necrosis factor-α (TNF-α) was elevated in both depression and mania and C-reactive protein (CRP) level was even increased in euthymia (25). In post-mortem BD brain samples, the microglia, astrocytes and macrophages were found to be involved in neuroinflammatory process (26). In addition, microglial activation was correlated with neuronal injury in the hippocampus (27). Reduced insula functional connectivity was related to the elevated level of IL-6 in drug- naïve BD patients (28). Elevated interferon-γ (IFN-γ) was related to the severity of mania while increased interleukin-1β (IL-1β) was associated with the severity of depression (9). All of these have indicated that the immune system plays a crucial role in BD. However, how immune dysregulation contributes to the onset of BD remains to be further elucidated.

### The Relationship Between the Inflammation and the KYN Pathway

Inflammatory factors can significantly shunt the TRP metabolism toward KYN pathway through upregulating the activity and expression of related rate-limiting enzymes. IFN-γ can independently induce the IDO activity. In addition, other inflammatory molecules, such as TNF-α and IL-1 can also synergistically enhance the IFN-induced IDO activity (29). The KMO transcript was increased via IL-1β stimulation and KMO expression was enhanced after LPS administration (30, 31). Furthermore, the activated immune state contributes to the impairment of BBB integrity (32), facilitating the transportation of TRP and KYN into the brain to provide original materials for the KYN pathway (18). Previous studies also showed that KYNA inhibited cytokine release to regulate the immune response through activating aryl hydrocarbon receptor while QA promoted the inflammatory response by inducing the production of proinflammatory mediators (33, 34). In this regard, the distinct metabolites of the KYN pathway may have opposites effects on immune responses.

In manic episodes, TNF-α level was positively connected with the IDO activity (represented by the KYN/TRP ratio) and neurotoxic production including 3-HK and QA. Besides, the KYNA/3-HK ratio was significantly reduced in and negatively correlated with IFN-γ in BD depressive subgroups (13). In patients with BD and/or schizoaffective disorder, Wurfel et al. found the KYNA/3-HK and KYNA/QA ratios were decreased and had negative correlations with CRP. However, the 3-HK, QA, 3-HK/KYN (a marker of KMO activity) and IDO activity were positively associated with CRP (35). As obesity is linked with chronic low-grade inflammation, an increased level of the KYN and a higher ratio of the KYN/TRP were also found in overweight, euthymic individuals with BD (36).

Therefore, immune dysfunctions in BD contribute to TRP metabolism toward the KYN pathway through activating activities of key enzymes, especially toward the neurotoxic metabolites.

### KYN Metabolites Modulate Neuroendocrine Transmission in BD

Once the KYN pathway has been activated, the signal transmissions of monoamine neurotransmitters and neuronal functions would be directly affected. For example, Williams et al. found that maternal inflammation shunted TRP metabolism away from the serotonin to the KYNA pathway, and thus potentially impaired the development of thalamocortical fibers in the brain of a new-born rabbit model (37).

KYNA, an endogenous glutamate receptor antagonist, inhibits the glycine-binding site of the NMDA receptor. It is demonstrated that the levels of glutamate would be reduced by 30–40% following even low dose administration of KYNA into the brain (38). Glutamate release was enhanced by the expression of the α7nAchR in the glutamatergic axon terminals, which could be recognized by KYNA in physiological concentrations, causing lower expression and activity of the α7nAchR (39). Additionally, KYNA can also modulate the dopaminergic neurotransmission via acting on the α7nAchR (40). The suppression of the N-type Ca^2+^ channels in sympathetic neurons could be partly attributed to the agonistic role of KYNA on an orphan G-protein-coupled receptor (GPR35) (41). Even though KYNA has also been regarded as a neuroprotective metabolite against the neurotoxic NMDA receptor, excessive KYNA accumulation would cause glutamatergic hypofunction and was associated with the psychotomimetic effects (42, 43).

In addition, QA accelerates the synaptosomal glutamate release, while inhibits the glutamate uptake by astrocytes (44). QA also has excitotoxic effects on GABAergic neurons and activates the glycine site of the NMDA receptor, which in turn reduces the level of the extracellular dopamine (45, 46). The prefrontal cortex (PFC), amygdala, hippocampus, as well as ventral striatum of the brain, are the preferred locations for QA-binding NMDA receptor subunits, where a greater excitotoxic burden is presented (47).

**Figure 3:**
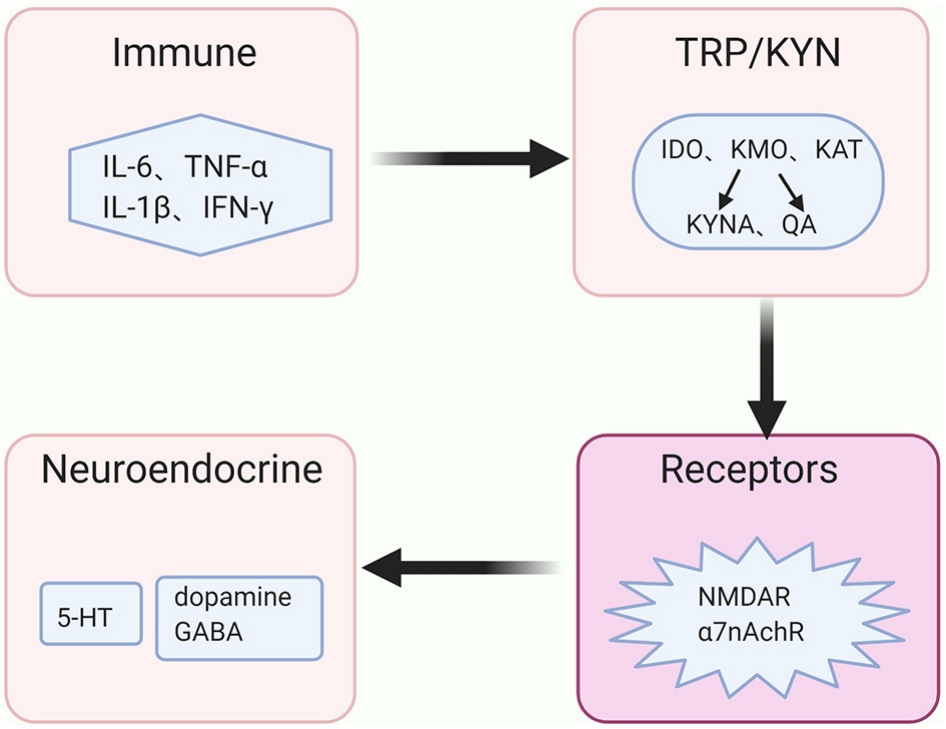
The immune-KYN-neuroendocrine network in BD. The abnormal immune system in bipolar disorder (BD) aberrantly activates the key enzymes' activities of the kynurenine (KYN) pathway, shifts the tryptophan (TRP) from serotonin (5-HT) toward kynurenine (KYN) pathway for metabolism, and causes the reduction of 5-HT. Levels and functions of other neurotransmitters, such as dopamine, γ-aminobutyric acid (GABA) as well as glutamate, rely on the metabolites of the KYN pathway in coordinating with distinct receptors, such as N-methyl-D aspartate (NMDA) receptor and non-competitively α7 nicotinic acetylcholine receptor (α7nAchR).

The above evidence demonstrates that inflammatory processes accelerate the KYN pathway metabolism in the brain and its metabolites eventually act on the receptors that are associated with the signals of neurotransmitters, such as dopamine, GABA and glutamate. Besides, serotonin plays a vital role in the pathogenesis of depressive behaviors via its effects on the 5-HT_1A_ receptor (48), and the release of the serotonin is reduced along with the KYN pathway activation Moreover, glutamatergic metabolism hypofunction may indicate an increased risk of depressed adolescents presenting mixed symptoms or developing BD (49). The high or low level of dopamine was thought to be related to the manic or depressive symptoms in BD, respectively (50). Therefore, we hypothesize that the KYN pathway may play a mediating role in the regulation of neuroendocrine signaling pathways and immune balance, which further participates in the pathogenesis of BD (Figure 3).

## Current Findings Indicating the KYN Pathway Alterations in BD

Up to present, alterations of the KYN pathway in BD have attracted adequate attention. Herein, we further reviewed current findings of the KYN metabolism dysfunctions in BD from perspectives of genetics, brain structural or functional imaging, as well as the relationships between the KYN metabolites and BD clinical symptoms (Table 1). In addition, some potential treatment strategies aiming at the KYN pathway have also been summarized.

**Table 1:** Demographic characteristics and findings of included studies on the KYN pathway in BD.

**References**	**Country**	**Sample size**	**Episode (In BD patients)**	**Gender (F/M)**	**Age (Mean ± SD)**	**Sample type**	**Main outcomes (In BD patients)**
van den Ameele et al. (13)	Belgium	BMD:32; BDD:35; HC:35	Manic Depressive	BMD:15F/17M; BDD:24F/11M; HC:19F/16M	BMD: 42.4 ± 12.7; BDD: 43.7 ± 9.7; HC: 42.7 ± 11.6	Serum	In BMD and BDD: KYNA↓ In BMD: TNF-α was positively associated with KYN, KYN/TRP, 3-HK and QA; In BDD: KYNA/3-HK↓; IFN-γ was negatively related with KYN/TRP, KYNA/3-HK and KYNA
Johansson et al. (69)	Sweden	BDI:10; CS:11; HC:12	Depressive Mixed Euthymic	BD:6F/4M; CS:6F/5M; HC:7F/5M	BD:40.6 ± 6.8; CS:44.4 ± 11.9; HC: 42.2 ± 12.7	Skin-derived fibroblasts	At baseline: KYNA↑; 3-HK↑; After cytokine treatments: KYNA↓; 3-HK↑
Wurfel et al. (35)	USA	BD:53; SZA:50; SZ:21; MDD:35; HC:92	–	BD:37F/16M; SZA:40F/0M; SZ:3F/18M; MDD:19F/16M; HC:59F/33M	BD: 40.2 ± 11.0; SZA: 39.0 ± 13.0; SZ: 38.9 ± 12.9; MDD: 38.8 ± 13.8; HC: 32.3 ± 10.4	Serum	KYNA↓; KYNA/QA↓; CRP was positively related with KYN/TRP, 3-HK, QA, and 3-HK/KYN while negatively with KYNA/3-HK and KYNA/QA
Olsson et al. (66)	Sweden	BD:31; HC:23	Euthymic	BD:0F/31M; HC:0F/23M	BD: 36.3 ± 9.2; HC: 33.1 ± 6.9	CSF	KYNA↑; KYNA was correlated with age.
Olsson et al. (67)	Sweden	BDI:55	Euthymic	BD: 34F/21M	BD(F): 37 ± 14; BD(M): 41 ± 14	CSF	KYNA↑ in euthymic BDI patients with a lifetime occurrence of psychotic features
Lavebratt et al. (51)	Sweden	Sample I: BD:34; SZ:36; HC:35 Sample II: BDI:493; HC:1,044 Sample III: BD:55	–	Sample I: BD:18F/16M; SZ:11F/27M; HC:11F/26M Sample II: BDI:284F/209M; HC:428/616M Sample III: BD:34F/21M	–	–	KMO mRNA↓ in PFC of BD patients with lifetime psychotic features. The KMO Arg^452^ allele was associated with the increased level of CSF KYNA and reduced lymphoblastoid and hippocampal KMO expression.
Poletti et al. (59)	Italy	BD (Depressed and Manic):72 HC:36	Manic Depressive	Depressed:35F/20M; Manic:8F/9M; HC:23F/13M	Depressed: 47.29 ± 9.86; Manic: 50.35 ± 11.92; HC: 43.86 ± 12.25	GM; WM; Serum	KYN↑; TRP↓; The KYN/TRP ratio was associated with variations in both GM and WM markers.
Poletti et al. (58)	Italy	BD:22; HC:15	Depressive	BD:14F/8M; HC:8F/6M	BD: 46.54 ± 13.66; HC: 27.20 ± 8.33	Serum; WM	KYNA↓; 5-HIAA↓; KYNA and 5-HIAA were related with WM integrity.
Miller et al. (60)	USA	BD:14; SZ:12; MDD:14; HC:14	–	–	–	Post-mortem WM	In BD: KYN↑; The density of glial cells (in both gray and white matter) stained for TDO2 was significantly increased.
Zhou et al. (73)	China	MDD:68 BD:16	Depressive	All patients: 45F/38M	–	Serum	At baseline: TRP↓; KYNA↓; KYNA/KYN↓; KYN/TRP↑; Ketamine responders: KYNA↑; KYNA/KYN↑; The elevated levels of KYNA and KYNA/KYN ratio were positively associated with the reductions in MADRS scores.
Platzer et al. (21)	Austria	BD:68; HC:93	Euthymic	BD:26F/42M; HC:57F/36	BD: F: 47.0 ± 13.5 M: 43.6 ± 14.3 HC: F: 39.4 ± 16.9 M: 38.1 ± 15.1	Serum	In males: KYN↑ and KYNA↑; The 3-HK /KYNA ratio was negatively correlated with the performance on the CVLT. The KYN/3-HK ratio was associated with performance on a sub-score of the CVLT.
Birner et al. (64)	Austria	BD:143; HC:101	Euthymic to mild depressive	BD:63F/80M; HC:61F/40M	BD:43.9 ± 13.3; HC: 40.3 ± 16.4	Serum	In BD: KYNA↓; 3-HK/KYN↑; 3-HK/KYNA↑
Sellgren et al. (68)	USA	BD:163; HC:114	–	BD:99F/64M; HC:62F/52M	–	CSF	KYNA↑;
Savitz et al. (61)	USA	BD:63 HC:48	Depressive	BD:51F/12M; HC:29F/19M	BD:38.8 ± 1.4; HC: 32.6 ± 1.5	Serum; Hippocampal volume; amygdalar volume	KYNA/QA↓; TRP↓ CRP was positively correlated with QA while negatively with KYNA/3-HK and KYNA/QA. KYNA/3-HK was positively associated with hippocampal volume and KYN/3-HK was significantly associated with total amygdalar volume in the BD group.
Kadriu et al. (22)	USA	BD:39; –	–	BD:23F/16M; –	BD: 45.92 ± 10.52; –	Serum	After treatment: IDO↓; QA/KYN↓; KYN↑; KYNA↑
Mukherjee et al. (70)	USA	BD:31; HC:28	–	BD:10F/21M; HC:16F/12M	BD: 36.10 ± 11.33; HC: 31.57 ± 10.33	Serum	TRP↓; The KYN/TRP ratio was associated with depressive severity while trended toward a negative association with mania symptoms in acutely symptomatic BD participants.
Reininghaus et al. (36)	Austria	BD: OW:54; NW:24; HC: OW:76; NW:80	Euthymic	–	BD: OW: 48.3 ± 13.1; NW: 43.7 ± 15.9; HC: OW: 37.0 ± 12.0; NW: 33.5 ± 10.9	Serum	KYN↑; KYN/TRP↑
Myint et al. (65)	Korea	BD:39; HC:80	Manic	BD:24F/15M; HC:40F/40M	BD: 37.6 ± 11.6 HC: 39.06 ± 8.75	Serum	TRP↓; KYNA↓

*For country, USA denotes United States of America. For gender, F and M denote female and male, respectively. SD, standard deviation; BD, bipolar disorder; BMD, bipolar manic disorder; BDD, bipolar depressive disorder; BDI, bipolar disorder type I; HC, healthy controls; CSF, cerebrospinal fluid; CS, chronic schizophrenia; SZ, schizophrenia; SZA, schizoaffective disorder; GM, gray matter; WM, white matter; KYNA, kynurenic acid; KYN, kynurenine; 3-HK, 3-hydroxykynurenine; QA, quinolinic acid; TRP, tryptophan; TDO, tryptophan 2,3-dioxygenase; 5-HIAA, 5-hydroxyindole acetic acid; KMO, kynurenine 3-monooxygenase; CRP, C-reactive protein; TNF-α, tumor necrosis factor-α; MADRS, Montgomery-Åsberg Depression Rating Scale; CVLT, California Verbal Learning Test; PFC, prefrontal cortex; OW, overweight; NW, normal weight. ↑: Up; ↓: Down*.

### Genetics

KMO has a high affinity for KYNA, and metabolizes most of KYN into 3-HK (51). Genetic variations, such as KMO Arg^452^ allele, affected its enzyme expression or activity and the reduced KMO function was correlated with the higher KYNA level in the post-mortem PFC from manic patients with psychotic features (51). A genetic polymorphism, i.e., rs9657182 in the *IDO1* gene enhanced the vulnerability to depressive symptoms along with the immune system activation in humans (52), whereas inhibition of IDO1 through genetic deletion or pharmacological manipulation could remove depressive phenotype in a mouse model (11).

IDO is vital for shunting KYN toward the microglial production of QA and may mediate depressive symptomatology (22). Excessive KYNA elevation was connected with the activation of the midbrain dopamine neurons (53, 54). Overactivation of the dopaminergic system may result in manic-like behaviors (55). Genetic variations affect the KYN metabolism, indicating the roles of genetic risk alleles in contributing to the vulnerability for BD.

### Brain Imaging

Neuroimaging studies have revealed unspecific brain structural changes in BD patients. The gray matter (GM) volumes and thickness in the cortex, especially in prefrontal and temporal cortices, were reduced in BD individuals and the volume of white matter (WM) ranging from the posterior corpus callosum to posterior cingulate cortex was decreased (56, 57). Further studies showed that the reduced KYNA level was negatively associated with the WM integrity in BD (58). A higher KYN/TRP ratio was negatively correlated with the volume of the right amygdala in the manic group and the left fusiform gyrus in the depressive patients (59). The KYN level was increased, while the KYNA/KYN ratio was decreased in the anterior cingulate in post-mortem patients with BD (60). Furthermore, the KYNA/3-HK was significantly positively correlated with the volumes of the amygdala and hippocampus (61). All of these findings showed the close relationship between the abnormal KYN pathway metabolites and brain architectures in BD patients.

Therefore, the alterations in the brain structures reflected the underlying processes of neurotoxicity and immune activation (62, 63). The neuroinflammation existing in BD could facilitate the production of neurotoxic metabolites via KYN pathway, which further contributed to the brain dysfunction and neuropathogenesis in BD.

### Relationships Between KYN Metabolites and BD Symptoms

Changes in KYN metabolites were linked to the clinical symptoms in BD individuals. Specifically, the ratios of 3-HK/KYN and 3-HK/KYNA were higher, while the KYNA level was decreased in BD patients with euthymia to mild depression compared to healthy controls (64). Furthermore, the lower level of the KYNA was also observed in both manic and depressive patient groups (13, 65). However, the higher level of KYNA was found in BD patients with previous psychotic features and males in euthymic states (66–69). Mukherjee et al. found that the KYN/TRP ratio was positively correlated with depressive severity but trended toward a negative correlation with manic symptoms in BD (70). Platzer et al. found that the 3-HK/KYNA ratio was higher in male patients and negatively connected with poorer verbal memory performance, indicating potential correlations between the KYN pathway and executive functions (21).

These findings provide valuable clues for the KYN pathway involved in the pathophysiology of BD, although the current results were inconsistent. Theoretically, KYN tends to be converted along the neurotoxic rather than the neuroprotective branch and the imbalance between these two branches plays a vital role in the etiology of BD. However, the mechanisms are complicated when it comes to the relationship between the KYN pathway metabolites and BD symptoms, predominantly including depressive or psychotic features, so that a concept of “double dissociation” has been proposed (35). Specifically, depression was associated with elevated levels of 3-HK and QA, which led to the inhibition of the NMDA receptor, and promoted glutamate release but reduced glutamate reuptake. The neuroprotective component KYNA was correlated with the psychotic features due to the striatal hyperdopaminergia and hypofunction of GABAergic interneurons. Considering the limitations of current studies, further researches are needed to fully clarify the pathophysiological mechanism of the KYN pathway on depressive, manic or psychotic features in BD.

### Potential Treatment Targets in BD

Considering the agonistic and antagonistic roles of KYNA and QA on the NMDA receptor, respectively, it is intriguing to explore treatment targets toward the KYN pathway in BD. As a non-competitive NMDA receptor antagonist, ketamine could alter the LPS-induced depressive-like behaviors through blocking the NMDA receptor activity (71). A single intravenous infusion of ketamine rapidly exerted rapid anti-depressive effect and improved suicidal ideations in type I or II bipolar depression (72). After intravenous infusions of ketamine, increases in the KYNA/KYN ratio and KYNA level were observed in responders and both were correlated with the Montgomery-Åsberg Depression Rating Scale (MADRS) scores' reduction compared to non-responders at 24-h and 13-day periods (73). AV-101, also named 4-chlorokynurenine, is a KYNA analog and selective antagonist of the NMDA receptor glycine binding site. It is an undergoing preliminary clinical trial for mood improvement and has received fast track designation by the Food and Drug Administration (74). Oral administration of a KYNA synthesis (KAT II) inhibitor promoted cognitive functions (75). KMO inhibitor could moderately and persistently increase the KYNA level in the brain to play neuroprotective roles (76). Therefore, modulation of the KYN pathway may be a promising treatment target for BD patients.

## Discussion

Our current knowledge on the role of the KYN pathway in BD is rapidly increasing. Growing evidence has indicated that the KYN pathway serves as a crucial regulator between the immune balance and neurotransmitter signaling in the pathogenesis of BD.

However, some specific questions need special attention and further investigations are warranted. Firstly, factors that contribute to the heterogeneous results about the KYN pathway in BD need to be controlled, including gender, age, race groups, and study methodologies. Moreover, the mood episodes of enrolled patients and the specific analysis aiming at subgroup or general population also affect the final findings. Polycentric and large samples research may help to untangle this dilemma.

Secondly, existing clinical studies mainly focus on the connections between the KYN metabolites and BD, but they could not draw causal conclusions. At present, it is still difficult to apply all preclinical research methods to humans through experimental manipulation of the KYN pathway. Therefore, multi-omic analysis that integrates host genomics, metabonomics, single cell sequencing, cytology, and brain network will be helpful for uncovering the complicated communicating networks of the KYN pathway in BD.

A meta-analysis investigated the alterations of the KYN metabolites in patients with unipolar depression and BD and found different results in these cases (77). Hence, clinical applications of the KYN pathway-related biomarkers for BD diagnosis still need further verification. Besides, explorations of novel therapeutic interventions and prognostic biomarkers derived from the KYN metabolites in BD are also needed. Cross-sectional and longitudinal studies may provide additional help to advance this field.

## Conclusion

In conclusion, convergent evidence has demonstrated that the immune system and the KYN pathway are changed in BD. Immune dysfunction could contribute to modulating the KYN pathway metabolism, which could further regulate the signals of the neurotransmitters, and finally affect the pathophysiological process of BD. The immune-KYN-neuroendocrine interaction in BD provides new clues for developing therapeutic targets.

## Author Contributions

PZ conducted literature searches and wrote the first draft of the manuscript. JL and SH gave substantial linguistic support. HH, XG, JJ, CX, LW, YF, and SH were involved in an intensive drafting and revision of the manuscript. All authors have read and approved the final manuscript.

## Conflict of Interest

The authors declare that the research was conducted in the absence of any commercial or financial relationships that could be construed as a potential conflict of interest.

## Publisher's Note

All claims expressed in this article are solely those of the authors and do not necessarily represent those of their affiliated organizations, or those of the publisher, the editors and the reviewers. Any product that may be evaluated in this article, or claim that may be made by its manufacturer, is not guaranteed or endorsed by the publisher.
